# Particulate Matter–Induced Airway Hyperresponsiveness Is Lymphocyte Dependent

**DOI:** 10.1289/ehp.0901461

**Published:** 2010-01-08

**Authors:** Vanessa Saunders, Patrick Breysse, Jennifer Clark, Alyssa Sproles, Melissa Davila, Marsha Wills-Karp

**Affiliations:** 1 Division of Immunobiology, Cincinnati Children’s Hospital Medical Center, University of Cincinnati College of Medicine, Cincinnati, Ohio, USA; 2 Department of Environmental Health Sciences, Johns Hopkins Bloomberg School of Public Health, Baltimore, Maryland, USA

**Keywords:** asthma, interleukins, outdoor air, particulate matter, pulmonary

## Abstract

**Background:**

Exposure to airborne particulate matter (PM), a major component of air pollution, has been associated with increases in both exacerbations of and hospitalizations for asthma. We have previously shown that exposure to ambient PM collected in urban Baltimore (AUB) induces airway hyperresponsiveness (AHR), eosinophilic and neutrophilic inflammation, and the recruitment of T cells. However, the mechanism(s) by which it induces these features of asthma remains unknown.

**Objective:**

We investigated whether T lymphocytes play a role in AUB-induced AHR.

**Methods:**

We compared the effects of AUB exposure on the allergic phenotype in wild-type (WT) BALB/c mice and in mice deficient in recombinase-activating gene-1 (*Rag1*^−/−^) that lack mature lymphocytes.

**Results:**

We found that exposure of WT mice to AUB induced AHR concomitant with increases in the numbers of bronchoalveolar lavage (BAL) fluid lymphocytes, eosinophils, neutrophils, and mucus-containing cells in the lungs of WT mice. Interestingly, we show for the first time that these effects were associated with significant elevations in interleukin (IL)-17A, IL-17F, and T-helper 2 cell (T_H_2) (IL-13, IL-5) cytokine levels in lung cells, as well as reductions in the levels of the suppressive cytokine IL-10. Interestingly, *Rag1*^−/−^ mice failed to develop AUB-induced AHR; however, AUB-induced BAL fluid cellularity, and mucus cell changes were only partially inhibited in *Rag1*^−/−^ mice.

**Conclusions:**

Taken together, our results suggest that AUB exposure increases the pathophysiological features of asthma via activation of lymphocyte-dependent pathways. These results provide a plausible biological mechanism for the strong association between PM exposure and the increased severity of asthma.

Asthma is a chronic inflammatory disease of the lung characterized by airway inflammation, airway hyperresponsiveness (AHR), and mucus hypersecretion. The current disease burden has reached epidemic proportions, and now an estimated 300 million people worldwide suffer with asthma (Global Initiative for Asthma 2008). Although the etiology of asthma is unknown, there is evidence that both genetic and environmental triggers contribute to disease. The recent rise in disease prevalence is unlikely to be explained by changes in the genetic makeup of society as a whole, which does not change dramatically in short time frames. Thus, changes in the environment are likely to be driving the marked increase in prevalence of this disease.

Environmental triggers of asthma include allergens, viruses, environmental tobacco smoke, and particulate matter (PM). Of these environmental triggers, several lines of evidence suggest that exposure to ambient PM may be associated with the increase in asthma morbidity. For example, numerous epidemiological studies have reported positive correlations between PM exposure and increased medication use, physician visits, and emergency department visits for asthma (Lipsett et al. 1997; Peel et al. 2005; Tolbert et al. 2000). Acute controlled exposures of healthy humans to PM have shown a wide variety of responses, from no significant effects on airway function or inflammation (Kongerud et al. 2006) to significant increases in cellular inflammation (Behndig et al. 2006; Ghio and Devlin 2001; Salvi et al. 1999, 2000; Samet et al. 2007; Schaumann et al. 2004). Despite wide variations in the study designs, sources, and composition of PM used in these studies, the most consistent findings have been that PM exposure increases neutrophils and inflammatory cytokines, such as interleukin(IL)-8 and IL-6, in bronchoalveolar lavage (BAL) fluid (Behndig et al. 2006; Ghio and Devlin 2001; Salvi et al. 1999, 2000; Samet et al. 2007; Schaumann et al. 2004), with some studies demonstrating increases in T lymphocytes (CD4^+^) in bronchial biopsies of healthy human volunteers (Salvi et al. 1999). Studies in animals have shown that direct instillation of biologically relevant sources of PM into the lungs of naive mice induces many of the pathophysiological features of asthma (Gavett et al. 1999; Ohta et al. 1999; Walters et al. 2001, 2002; Wang et al. 2008). Although many hypotheses have been put forth to explain the ability of PM to directly induce or exacerbate asthmalike symptoms, to date the exact mechanisms underlying the adverse pulmonary effects of PM are not well understood.

Because numerous studies in animal models have shown that exposure to other environmental triggers such as allergens (Cohn et al. 1998; Corry et al. 1998; Gavett et al. 1994) and ozone (Chen et al. 1995) induce AHR through a T cell–dependent process, and because PM has been shown to drive T-cell cytokine production *in vivo* (Walters et al. 2001; Wang et al. 2008) and in culture systems (Porter et al. 2007; Williams et al. 2007), we hypothesized that PM-induced AHR and airway inflammation occur through a lymphocyte-dependent process. Thus, the objective of the present study was to directly explore the role of lymphocytes in the development of PM-induced AHR and airway inflammation. To this end, we compared the effects of ambient PM collected in urban Baltimore (AUB) on airway reactivity and allergic inflammation in wild-type (WT) BALB/c mice and in mice deficient in recombinase-activating gene 1 (*Rag1*^−/−^) that lack mature lymphocytes. We show that AUB induced AHR, pulmonary inflammation, and mucus metaplasia, concomitant with increases in both T-helper 2 cell (T_H_2) and T_H_17 cytokine production. In marked contrast, *Rag1*^−/−^ mice do not develop AHR or T_H_2/T_H_17 cytokine production after AUB exposure. However, AUB-induced increases in BAL fluid and tissue inflammation as well as mucus production were only partially lymphocyte dependent. Collectively, our results demonstrate that pulmonary exposure to a real-world source of PM induces the recruitment and activation of T cells leading to the induction of the pathophysiological features of asthma.

## Materials and Methods

### Mice

Male and female C.129S7(B6)-Rag1tm1Mom/J (*Rag1*^−/−^) mice and BALB/c (WT) control mice (9–10 weeks of age; Jackson Laboratories, Bar Harbor, ME) were housed in an environmentally controlled specific pathogen–free facility at Cincinnati Children’s Hospital Medical Center. The mice received access to food and water *ad libitum*. Mice were treated humanely and with regard for alleviation of suffering in accordance with the Cincinnati Children’s Hospital Institutional Animal Care and Use Committee.

### PM exposure

Ambient PM was collected from a sixth floor window in urban Baltimore during the months of March through May in 2005 using a high-volume cyclone collector with a theoretical cut-point of 0.85 μm aerodynamic diameter when operated at a flow rate of 0.6 m^3^/min (Walters et al. 2001). Mice were anesthetized with an intraperitoneal injection of a mixture of ketamine (45 mg/kg) and xylazine (8 mg/kg) and exposed to either phosphate-buffered saline (PBS) or AUB (0.5 mg in a 50-μL volume of PBS) on days 0, 3, and 6 of the study by intratracheal instillation.

### Airway responsiveness measurements

We evaluated airway responsiveness to intravenous acetylcholine 24 hr after the final AUB exposure as previously described (Lewkowich et al. 2005). Briefly, mice were anesthetized, intubated, and respirated at a rate of 120 breaths/min with a constant tidal volume (0.2 mL) and paralyzed with 25 mg/kg decamethonium bromide 72 hr after final allergen challenge. After a stable baseline was achieved, 50 mg/kg acetylcholine was injected into the inferior vena cava, and dynamic airway pressure (cm H_2_O/sec) was followed for 5 min.

### Determination of cellularity and chemokine levels in BAL fluid

Lungs were lavaged three times with a 1.0-mL aliquot of cold Hanks’ balanced salt solution. Recovered lavage fluid (70–80%) was centrifuged at 300 × *g* for 8 min, and the cell pellet was resuspended in 1.0 mL 10% fetal bovine serum in PBS. Slides were prepared by cytocentrifugation and stained with Diff-Quik (Dade Behring, Dudingen, Switzerland). Total and cell differential counts were determined in BAL fluid using morphologic criteria under a light microscope with the evaluation of > 500 cells/slide. BAL fluid chemokine levels were determined by enzyme-linked immunosorbent assay (ELISA) (R&D Systems, Minneapolis, MN).

### Lung T-cell identification and cytokine measurements

Whole lungs were perfused with ice-cold PBS, removed, minced, placed in 6 mL RPMI 1640 containing 0.5 mg/mL collagenase (Liberase CI; Roche Diagnostics, Indianapolis, IN) and 0.5 mg/mL DNase I (Sigma-Aldrich, St. Louis, MO), and incubated at 37°C for 45 min. Single-cell suspensions were pelleted and stained with allophycocyanin-conjugated anti-mouse CD4 (L3T4) and fluorescein isothiocyanate–conjugated anti-mouse T-cell receptor β (TCRβ) for flow cytometric analysis (eBioscience, San Diego, CA) using a FACSVantage SE flow cytometer (BD Biosciences, Franklin Lakes, NJ). Analysis was performed using FLowJo software (version 3.2; Tree Star, Inc., Ashland, OR). For T-cell cytokine determination, lung cells (250,000) were cultured in media or concanavalin A (ConA; 5 μg/mL) for 72 hr, and cytokine levels were measured by ELISA.

### Histological examination of lung sections

To assess the effects of AUB on airway inflammation and mucus cell content in the airway wall, lungs were excised and fixed in 10% formalin, washed in methanol, dehydrated, embedded in paraffin, and cut into 5-μm sections. Sections were mounted on slides and stained with hematoxylin and eosin or periodic acid-Schiff (PAS). Slides were read in a blinded fashion and scored according to the following scale: 0, no inflammation; 1–1.99, 1–25% inflammation of section; 2–2.99, 26–50% inflammation of section; 3–3.99, 51–75% inflammation of section; 4–4.99, 76–100% inflammation of section. We counted the number of PAS-positive cells per section using a light microscope, and results are presented as mean ± SE for four sections per mouse lung.

### Measurement of serum IgE levels

Immediately after AHR measurements, terminal blood was collected from the posterior vena cava. Total serum IgE levels were measured by ELISA using matched antibody pairs (BD Pharmingen, Franklin Lakes, NJ).

### Statistical analysis

We determined differences between multiple groups using one-way analysis of variance, with Tukey multiple comparison post-test comparisons. To compare the two groups, we used Student’s *t*-test (GraphPad Prism; GraphPad Software Inc., La Jolla, CA). Significance was assumed at *p* < 0.05.

## Results

### AUB-induced AHR is lymphocyte dependent

**Figure 1:**
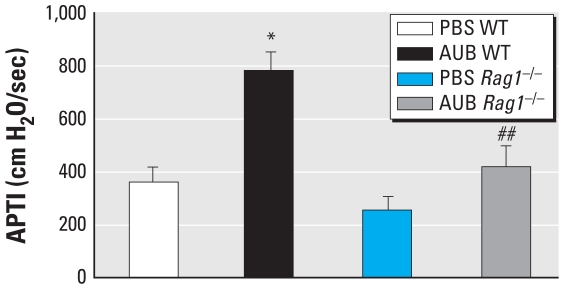
AUB-induced AHR is lymphocyte dependent, as determined by airway responses of AUB-exposed WT and *Rag1*^−/−^ mice to acetylcholine (50 mg/kg). Results are shown as the mean ± SE of the time-integrated change in airway pressure (APTI); *n* = 7–10 mice/group. *p < 0.0001 compared with PBS WT. ^##^*p* < 0.01 compared with AUB WT.

To assess the contribution of lymphocytes to the development of AUB-induced AHR, we compared the airway responses of WT and *Rag1*^−/−^ mice to the cholinergic agonist acetylcholine. In WT mice exposed to AUB, we observed a significant increase in AHR compared with PBS controls (*p* < 0.0001; Figure 1). In contrast, no significant increases in AHR were seen in AUB-exposed *Rag1*^−/−^ mice compared with the AUB-exposed WT mice. Thus, airway responses in both WT and *Rag1*^−/−^ mice exposed to AUB were significantly different (*p* < 0.01). Lymphocytes also contributed to the baseline airway response to acetylcholine because the response was lower in PBS-challenged *Rag1*^−/−^ mice compared with the PBS-exposed WT controls, although the observed difference did not achieve statistical significance.

### AUB induction of mucus is partially lymphocyte dependent

**Figure 2:**
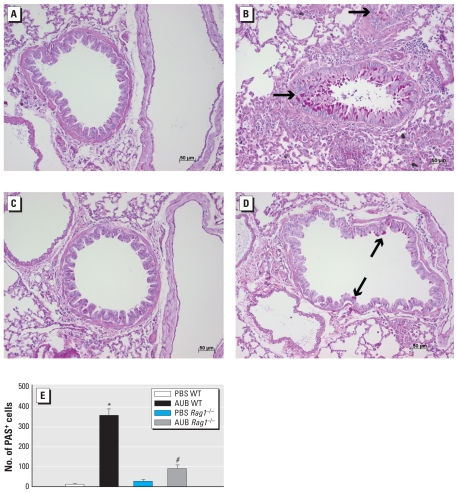
AUB induced a partially T-cell–dependent increase in mucus cells, as shown by PAS staining in lung sections from PBS-exposed (*A* and *C*) and AUB-exposed (*B* and *D*) WT (*A* and *B*) and *Rag1*^−/−^ mice (*C* and *D*). Arrows indicate PAS-positive (PAS^+^) staining. (*E*) The number of PAS^+^ cells (mean ± SE) in airways in four sections per mouse lung (*n* = 4 mice/group). *p < 0.0001 compared with PBS WT. ^#^*p* < 0.0001 compared with AUB WT.

Because mucus metaplasia is a consistent feature of allergic asthma, we assessed the effects of AUB exposure on mucus production as assessed by PAS staining. We observed significant increases in the numbers of airways containing PAS-positive mucus cells in the lungs of AUB-exposed WT (Figure 2B) and *Rag1*^−/−^ (Figure 2D) mice compared with their respective PBS controls (Figure 2A,C). The numbers of PAS-positive cells in AUB-exposed *Rag1*^−/−^ mice were significantly higher than those in their PBS controls, but significantly lower than those observed in the WT AUB-exposed mice (*p* < 0.0001; Figure 2E).

### AUB-induced BAL fluid inflammation is partially lymphocyte dependent

**Figure 3:**
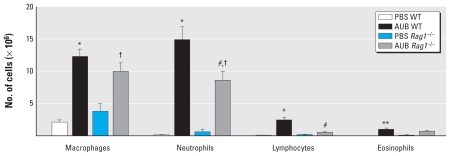
Populations of macrophages, neutrophils, lymphocytes, and eosinophils in Bal fluid of WT and *Rag1*^−/−^ mice after AUB exposure. Values are mean ± SE; *n* = 7–10 mice/group. *p < 0.0001, and **p < 0.01 compared with PBS WT. ^#^*p* < 0.0001 compared with AUB WT. ^†^*p* < 0.0001 compared with PBS *Rag1*^−/−^.

**Figure 4:**
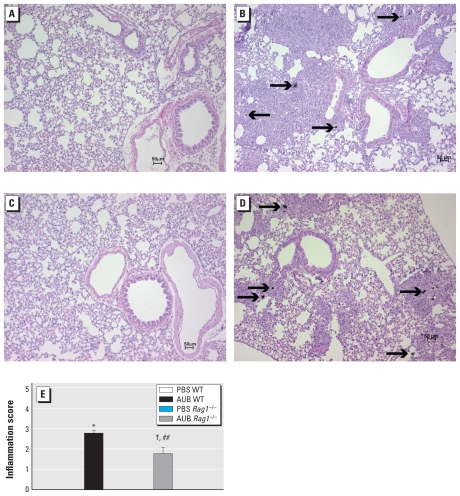
AUB-induced lung inflammation is partially lymphocyte dependent, as shown by hematoxylin and eosin staining of WT (*A* and *B*) and *Rag1*^−/−^ (*C* and *D*) mice exposed to either PBS (*A* and *C*) or AUB (*B* and *D*). Arrows indicate AUB particles. (*E*) Degree of inflammation scored according to an arbitrary scale defined in “Materials and Methods.” Values are mean ± SE; *n* = 8 independent sections/group. *p < 0.0001 compared with PBS WT. ^##^*p* < 0.01 compared with AUB WT. ^†^*p* < 0.0001 compared with PBS *Rag1*^−/−^.

To determine whether AUB induces allergic airway inflammation in a lymphocyte-dependent manner, we compared the cellularity of the BAL fluids from *Rag1*^−/−^ and WT mice after PBS or AUB exposure. In PBS controls of both strains of mice, most of the cells in the BAL fluid were primarily alveolar macrophages and neutrophils (Figure 3). We observed significant elevations in the numbers of macrophages, neutrophils, and eosinophils in BAL fluid from AUB-exposed WT mice. We also observed increases in each cell type in AUB-exposed *Rag1*^−/−^ mice. However, the levels of neutrophils and lymphocytes were significantly lower in the BAL fluid from AUB-exposed *Rag1*^−/−^ mice compared with AUB-exposed WT mice, whereas we found no significant differences in the numbers of macrophages between *Rag1*^−/−^ and WT PBS controls. Although eosinophils were elevated in AUB-exposed *Rag1*^−/−^ mice, the difference was not statistically significant. Although *Rag1*^−/−^ mice are devoid of T and B lymphocytes, we observed a small number of cells in the BAL fluid with morphological characteristics of lymphocytes. These are most likely natural killer (NK) cells, because NK cell numbers are increased in naive *Rag1*^−/−^ mice (Grundy and Sentman 2006). Consistent with the inflammatory patterns seen in BAL fluid after AUB exposure, we observed widespread perivascular and peribronchial inflammation in the lungs of both WT and *Rag1*^−/−^ mice compared with their PBS-exposed controls (Figure 4). Consistent with the partial effect of *Rag1* deficiency on AUB-induced increases in BAL fluid cellularity, the degree of AUB-induced inflammation in lung sections from *Rag1*^−/−^ mice was significantly reduced compared with that seen in WT mice (*p* < 0.05; Figure 4E) but still significantly higher than in their PBS-exposed controls. Based on cell morphology, the inflammatory foci consisted primarily of neutrophils and macrophages. Of note, we detected AUB particles in the sections presumably engulfed by macrophages (Figure 4B,D). Taken together, these results suggest that AUB exposure induces a marked cellular infiltration of the mouse lung, which is only partially lymphocyte dependent.

### AUB induces the recruitment of CD4^+^ T cells

**Figure 5:**
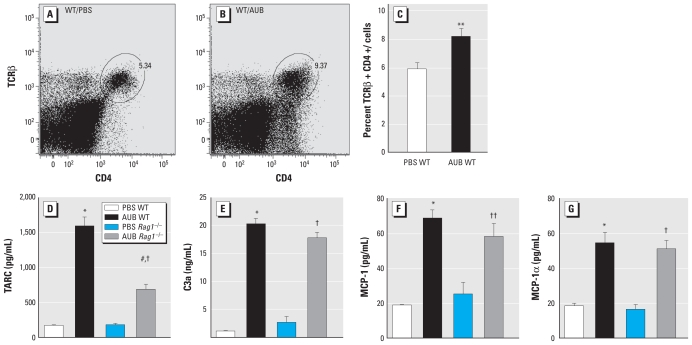
AUB increases CD4^+^ T-cell recruitment concomitant with increases in chemokine production in the lung as detected by flow cytometry. Representative flow cytometry plots of CD4^+^ and TCRβ^+^ cells in BAL fluid of WT mice exposed to PBS (*A*) or AUB (*B*). (*C*) Quantification of the percentage of CD4^+^ T cells (mean ± SE) in lungs of WT mice treated with PBS or AUB (*n* = 7–10 mice/group). (*D*–*G*) Levels (mean ± SE) of TARC (*D*), C3a (*E*), MCP-1 (*F*), and MIP-1α (*G*) measured in BAL fluid of AUB- or PBS-exposed WT or Rag^−/−^ mice (*n* = 7–10 mice/group). **p < 0.01 compared with PBS WT. *p < 0.0001 compared with PBS WT. ^#^*p* < 0.0001 compared with AUB WT. ^†^*p* < 0.0001. ^††^*p* < 0.01 compared with PBS *Rag1*^−/−^.

To gain additional insight into the type of T lymphocytes recruited by AUB exposure, we assessed the effects of AUB exposure on the numbers of CD4^+^ TCRβ^+^ cells by flow cytometric analysis of whole-lung cell digests. Our results revealed that AUB exposure induced significant increases in the percentage of conventional CD4^+^ TCRβ^+^ T cells in the lungs of WT mice (Figure 5A,B). As expected, we found no detectable CD4^+^ T cells in *Rag1*^−/−^ animals (data not shown).

To begin to understand the mechanism(s) of AUB-induced CD4^+^ T-cell subset recruitment, we assessed the effects of AUB exposure on the levels of several chemoattractants [thymus and activation regulated chemokine CCL17 (TARC), complement factor 3a (C3a), monocyte chemotactic protein-1 (MCP-1), and macrophage inflammatory protein 1α (MIP-1α)], all of which are known to contribute to T cell–mediated inflammation (Gu et al. 2000; Kawasaki et al. 2001; Ritz et al. 2004; Walters et al. 2002). We found that AUB induced a significant increase in the levels of each of these chemokines in BAL fluid (Figure 5D–G). However, only TARC production appeared to be dependent on the presence of lymphocytes because it was significantly lower in AUB-exposed *Rag1*^−/−^ mice than in AUB-exposed WT mice (Figure 5D). These results demonstrate that AUB exposure likely initially induces the recruitment of inflammatory cells into the lungs through the production of chemoattractants such as C3a, MCP-1, MIP-1α, and TARC, which are likely produced by either airway epithelial cells or monocyte/macrophage populations.

### AUB induces T-cell cytokine production in WT mice

**Figure 6:**
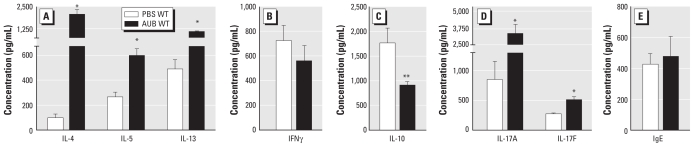
Effect of AUB on cytokine and IgE production in lung T cells. (*A*–*D*) Cytokine production from ConA-restimulated lung cells showing the T_H_2 cytokines IL-4, IL-5, and IL-13 (*A*), the T_H_1 cytokine IFNγ (*B*), the Treg cytokine IL-10 (*C*), and the T_H_17 cytokines IL-17A and IL-17F (*D*). (*E*) Total serum IgE levels in AUB-exposed WT mice. Values are mean ± SE; *n* = 7–10 mice/group. *p < 0.0001, and **p < 0.01 compared with PBS WT.

To assess the nature of the lymphocyte cytokine response to AUB, we measured the levels of cytokines associated with T_H_2, T_H_17, T_H_1, and regulatory T-cell (Treg) effector cell function from lung homogenates restimulated *in vitro* with ConA. Restimulated lung cells from AUB-exposed WT mice produced a higher level of the T_H_2 cytokines IL-4, IL-5, and IL-13 than did their PBS-exposed controls (Figure 6A), whereas we found no significant induction of the T_H_1-associated cytokine interferon-γ (IFNγ) (Figure 6B). Consistent with increased inflammation in the lungs and the increased number of lymphocytes in BAL fluid after AUB exposure, we found a significant reduction in the immunosuppressive cytokine IL-10 in cells from AUB-exposed WT mice (Figure 6C). AUB-exposed WT mice also had significantly higher levels of the T_H_17-associated cytokines IL-17A and IL-17F than the PBS-exposed controls (Figure 6D). As expected, lung cells from *Rag1*^−/−^ mice did not respond to ConA stimulation (data not shown).

To determine whether AUB exposure induces allergic sensitization, we measured total serum IgE levels from AUB-exposed WT mice. In these mice, we found no increase in total serum IgE levels compared with PBS-exposed controls (Figure 6E). These results suggest that acute exposures to AUB do not promote atopy and that the AHR and inflammatory responses observed in AUB-exposed mice at the time point assessed do not depend on IgE-driven processes.

## Discussion

In the present study, mice exposed to AUB showed marked increases in airway responsiveness to cholinergic stimuli, concomitant with an increase in eosinophilic and neutrophilic inflammation, CD4^+^ T-cell recruitment, and mucus cell metaplasia. These findings are consistent with epidemiological studies linking PM exposure and recent increases in asthma prevalence and morbidity. Moreover, our results support previous human PM exposure studies (Behndig et al. 2006; Ghio and Devlin 2001; Salvi et al. 2000; Samet et al. 2007; Schaumann et al. 2004) and studies in mouse models from our group and others showing that different sources of ambient PM [AUB, fly ash, diesel exhaust particles (DEPs)] can directly induce the pathophysiological features of asthma (Gavett et al. 1999; Ohta et al. 1999; Walters et al. 2001, 2002), as well as enhance immune responses to other allergens (Gavett et al. 1999; Fernvik et al. 2002; Wang et al. 2008).

Our results demonstrate that AUB-induced AHR is dependent upon lymphocytes because AHR is significantly attenuated in mice lacking mature lymphocyte populations (*Rag1*^−/−^ mice). These results are consistent with other reports showing that the development of AHR in response to other environmental triggers of asthma, such as allergens (Cohn et al. 1998; Corry et al. 1998; Gavett et al. 1994), airborne oxidants (Chen et al. 1995), and irritants (Garssen et al. 1990; Matheson et al. 2001), are dependent on T lymphocytes. Although the development of AHR in response to AUB depended on lymphocytes, the AUB-induced influx of inflammatory cells into the lungs, as assessed by both BAL fluid and histological examination of lung sections, was only partially dependent on lymphocytes. This apparent disassociation between inflammation and AHR is in agreement with previous reports from our laboratory indicating that airway inflammation does not correlate with the development of antigen- or PM-induced AHR (Lewkowich et al. 2005; Walters et al. 2002). Likewise, AUB-induced mucus cell metaplasia was only partially abrogated in *Rag1*^−/−^ mice. Because previous studies have shown the importance of CD4^+^ T-cells and the T_H_2 cytokine IL-13 (Wills-Karp et al. 1998) in mucus production after antigen exposure, these results are somewhat surprising. The results may suggest that either AUB directly induces mucus cell changes in the airway epithelium or that other innate immune cells such as neutrophils contribute to the induction of this response in *Rag1*^−/−^ mice. Indeed, several studies have implicated neutrophil-derived mediators (neutrophil elastase) in mucus production (Shao and Nadel 2005).

Because T_H_2 cytokines have been closely associated with the development of antigen-induced AHR, we examined the cytokine profile in the lungs of mice exposed to AUB. We found that AUB induced a marked influx of CD4^+^ T-cells into the lungs and elevations in the levels of both T_H_2 (IL-4, IL-5, IL-13) and T_H_17 (IL-17A, IL-17F) cytokines, concomitant with a reduction in the Treg cytokine IL-10. We saw no significant changes, compared with baseline levels, in the T_H_1 cytokine IFNγ. Taken together, these results suggest that AUB induces allergic inflammation both by suppressing tolerogenic immune responses (IL-10) and by inducing a T_H_2/T_H_17 mixed immune response in the airways.

Here, we show for the first time that AUB-induced AHR is associated with the induction of a mixed T_H_17/T_H_2 cytokine response in the lung. Our observation is consistent with recent studies in human asthmatics and in animal models of ozone- and allergen-induced AHR implicating IL-17 in the development and progression of allergic asthma. Specifically, recent studies have shown that IL-17A levels in asthmatics correlate with the incidence of AHR and severity of disease (Barczyk et al. 2003; Chakir et al. 2003). Likewise aerosolized pollutants (organic dust and ozone) have been shown to induce IL-17A in human BAL cells (Ivanov et al. 2005) and in the mouse lung (Pichavant et al. 2008). Indeed, recent studies have demonstrated a primary role for IL-17 in ozone-induced AHR because both antibody blockade of IL-17 and genetic deficiency in the IL-17R protect against ozone-induced AHR (Pichavant et al. 2008). Studies of IL-17 in allergen-induced models of AHR suggest a more complex picture, with some studies showing that IL-17 plays an important role in allergen-driven AHR (Nakae et al. 2002; Wakashin et al. 2008; Wilson et al. 2009), whereas others have shown that either IL-17 does not play a role at all (Hellings et al. 2003) or that it can either stimulate or inhibit the development of allergic inflammation depending on the timing of IL-17 blockade (Schnyder-Candrian et al. 2006). Despite the evidence implicating IL-17 in AHR, it alone does not appear to be sufficient to induce AHR; however, IL-17 has been shown to synergize with T_H_2 cytokines (Wakashin et al. 2008; Wilson et al 2009). Although it’s role in AHR is not known, IL-17 is a potent stimulator of neutrophil recruitment and activation, and IL-17–dependent AHR has recently been shown to be neutrophil dependent (Wilson et al. 2009). A role for neutrophils in the induction of AHR in our model is suggested by the fact that AUB induced a significant influx of neutrophils into the mouse lung and that the numbers of neutrophils in the BAL fluid were reduced in *Rag1*^−/−^ mice concomitant with suppression of AHR. Taken together, these results suggest that the development of AHR in response to airway delivery of antigens/pollutants may be dependent upon the synergistic actions of T_H_2 and IL-17.

The source of T_H_2 and T_H_17 cytokines appears to be lymphocytes because both baseline and AUB-stimulated cytokine levels are absence in *Rag1*^−/−^ mice. Specifically, although not proven, we propose that the cells producing these cytokines are CD4^+^ T cells because we observed a marked influx of CD4^+^ T cells after AUB exposure (Figure 4B,E), which was absent in *Rag1*^−/−^ mice. However, a contribution by other lymphocyte populations cannot be ruled out. Indeed, recent studies suggest that NK T cells may contribute to the development of AHR induced by ozone (Pichavant et al. 2008) through their ability to recognize lipid antigens and produce cytokines soon after exposure. NK T cells likely do not play a role in our studies because *Rag1*^−/−^ mice do not have mature NK T cells. However, NK cells may play a role because the development of these cells is up-regulated in *Rag1*^−/−^ mice (Grundy and Sentman 2006). These cells may account for the lymphocyte-like populations we identified morphologically in the BAL fluid, and they may be responsible for the lymphocyte-independent production of IL-5 and recruitment of inflammatory cells we observed in the lungs of *Rag1*^−/−^ mice.

The mechanisms by which PM activates lymphocytes are currently unknown. We have previously reported that AUB contains a variety of potentially biologically active components such as endotoxin, metals, and polyaromatic hydrocarbons (PAHs) (Walters et al. 2001). Recent studies suggest that pollutants such as ozone activate lymphocytes in the mouse lung (Pichavant et al. 2008). Although it had been thought that substances such as ozone damage the airways through production of free radicals, leading to the presentation of altered self-proteins (Ciencewicki et al. 2008), recent studies suggest that ozone may also induce airway inflammation through toll-like receptor 4 (TLR4)-mediated processes (Kleeberger et al. 2000). We previously reported that AUB contains low levels of endotoxin (Walters et al. 2001); thus, the activation of immune responses in our model may be at least partially TLR4 dependent. Alternatively, the oxidative potential of transition metals (copper, manganese, zinc) and PAHs contained in our PM source may also drive AUB-induced T-cell activation. Substantial evidence suggests that metals and oxidative stress play a significant role in the strong epidemiological association between indices of allergic airway disease and PM exposure in epidemiological studies conducted both in the Utah Valley (Ghio and Devlin 2001; Pope 1989) and in Germany (Schaumann et al. 2004). PAHs are also thought to be strong inducers of oxidative stress because the ability of DEPs containing high levels of PAHs to enhance ovalbumin sensitization in mice is inhibited by pretreatment of mice with thiol antioxidants (Li et al. 2009; Whitekus et al. 2002). The water-soluble fraction of AUB does not contribute to its ability to induce AHR (Walters et al. 2001), thus, the organic fraction of AUB containing numerous PAHs may play a significant role in T-cell activation in our model. Each of these components is likely to activate T cell–mediated immune responses through effects on dendritic cell function rather than direct effects on T cells, because DEPs collected in urban Baltimore do not directly activate T cells (Porter et al. 2007). In contrast, AUB has been shown to directly activate dendritic cells in an oxidant-dependent manner (Williams et al. 2007). Taken together these results suggest that AUB activates dendritic cell/T-cell activation through multiple additive or synergistic effects driven by the individual components of real-world ambient air PM.

## Conclusion

Our studies demonstrate that exposure of the mouse lung to real-world ambient PM directly induces several features of asthma, concomitant with the activation of an adaptive immune response characterized by the recruitment and activation of CD4^+^ T lymphocytes. The induction of a T_H_2/T_H_17-skewed cytokine environment in the lung may directly drive asthmatic symptoms, as well as lead to the sensitization to or enhancement of ongoing immune responses to heterologous antigens in susceptible individuals. These studies provide a plausible biological mechanism for the strong association between PM exposure and the increase in asthma morbidity.
